# Cortical Brain Activation During Robot‐Assisted Gait in Humans With Acute and Chronic Spinal Cord Injury: A Functional Near‐Infrared Spectroscopy Study

**DOI:** 10.1111/ejn.70459

**Published:** 2026-03-10

**Authors:** Ana Rita C. Donati, Daniel Boari Coelho, João Ricardo Sato, Felipe Fregni, Linamara Rizzo Battistella

**Affiliations:** ^1^ Instituto de Medicina Física e Reabilitação, Hospital das Clínicas HCFMUSP, Faculdade de Medicina Universidade de São Paulo São Paulo São Paulo Brazil; ^2^ Associação de Assistência à Criança Deficiente (AACD) São Paulo São Paulo Brazil; ^3^ Department of Biomedical Engineering Federal University of ABC São Bernardo do Campo São Paulo Brazil; ^4^ Departamento de Medicina Legal, Bioética, Medicina do Trabalho e Medicina Física e Reabilitação, Faculdade de Medicina Universidade de São Paulo São Paulo São Paulo Brazil; ^5^ Center for Mathematics, Computation, and Cognition Federal University of ABC São Bernardo do Campo São Paulo Brazil; ^6^ Neuromodulation Center Spaulding Rehabilitation Hospital, Harvard Medical School Boston Massachusetts USA

**Keywords:** motor cortex, neurorehabilitation, walking

## Abstract

Most treatments being developed to regain motor function following spinal cord injury (SCI) presuppose that brain motor functions remain intact. To examine this assumption, this study aims to analyze residual neurological functions during assisted robotic gait in individuals with SCI comparing blocks (gait × resting), time after SCI (acute × chronic), injury level (paraplegic × tetraplegic), and ASIA scale (ASIA C × D). The hemodynamic functions were analyzed using functional near‐infrared spectroscopy (fNIRS) in 23 individuals (11 acute, 12 chronic; ASIA Impairment Scale grade C: 10, D: 13; paraplegia: 15, tetraplegia: 8) while performing an assisted robotic gait task (Lokomat). Brain areas analyzed included supplementary motor area (SMA), dorsolateral prefrontal cortex (DLPFC), primary motor cortex (M1), and primary somatosensory cortex (S1). Blocks (robotic gait × resting), acute × chronic, paraplegic × tetraplegic, and ASIA C × ASIA D groups were compared. For the block comparison, there was a significant difference in SMA and M1, with higher oxyhemoglobin values in the robotic gait task compared to resting. For the comparison between groups, there was a significant difference in M1, with higher oxyhemoglobin values in the chronic group compared to the acute group. The individuals with paraplegia exhibited greater activity in M1 than those with tetraplegia during the robotic gait task. These results demonstrate the plasticity and adaptability of brain motor cortex areas even during the chronic phase after SCI. The brain motor cortex activity during a walking motor task reinforces the importance of analyzing residual neurological function after SCI.

## Graphical abstract

Panel (A) shows the experimental setup, illustrating the positioning of the individual with spinal cord injury within the robotic gait device. Panel (B) presents the experimental flowchart. Panel (C) shows the mean values (standard deviation) of the hemodynamic response to oxygenated hemoglobin in the supplementary motor area (SMA), dorsolateral prefrontal cortex (DLPFC), primary motor cortex (M1), and primary somatosensory cortex (S1) for the robotic gait (black) and resting (red) blocks.

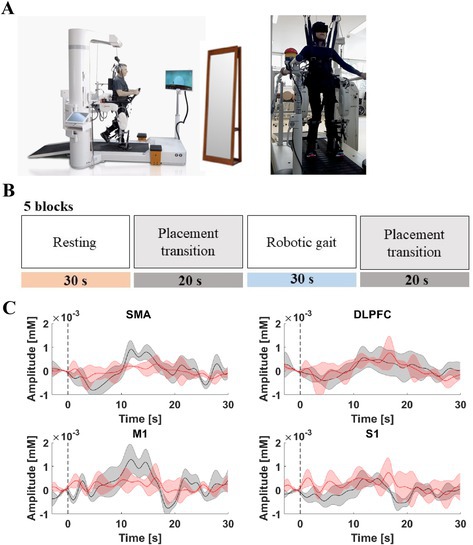

**Abbreviations**
AIS: ASIA Impairment Scale
ASIA: American Spinal Injury Association
BWST: body‐weight support system on a treadmill
deoxy‐Hb: deoxyhemoglobin
DLPFC: dorsolateral prefrontal cortex
fNIRS: functional near‐infrared spectroscopy
fOLD: fNIRS Optodes' Location Decider
GLM: general linear model
M1: primary motor cortex
oxy‐Hb: oxyhemoglobin
S1: primary somatosensory cortex
SCI: spinal cord injury
SMA: supplementary motor area

## 1 Introduction

Epidemiological data provide alarming parameters, indicating that approximately 250,000 to 500,000 people become victims of spinal cord injuries (SCIs) worldwide every year (Biering‐Sorensen et al. 2011). There is a growing interest in unraveling the real impact of SCI on human neurological functions. The gold standard clinical classification of SCI, the ASIA Impairment Scale (AIS), developed by the American Spinal Injury Association (ASIA), may not sufficiently identify the residual neurological function in all individuals. Research has shown that the complexity of SCI extends beyond the simplistic interaction of spinal tracts (Dimitrijevic 1987, 1988; Sherwood et al. 1992). The concept of discomplete injury refers to a clinically complete injury (ASIA A) but with neurophysiological evidence of residual brain influence on spinal cord function below the lesion. Additionally, a post‐mortem anatomical‐pathological study (Kakulas et al. 1998) revealed anatomical spinal cord continuity in individuals clinically diagnosed with complete injury, suggesting that clinical neurological evaluations could be more accurate when paired with a comprehensive analysis of SCI's impact on neurophysiological brain activity. In this context, the use of functional near‐infrared spectroscopy (fNIRS) may contribute to more precisely detecting residual neurological functions, standardizing a method to find cortical brain responses in subjects with SCI, and enhancing neurological rehabilitation with a functional purpose. This non‐invasive, low‐cost neuroimaging technology has significant potential for clinical applications.

Recent studies have highlighted the effectiveness of assisted robotic gait devices in improving lower extremity motor function and daily living activities in individuals with SCI (Stampacchia et al. 2016; Ramanujam, Cirnigliaro, et al. 2018; Ramanujam, Momeni, et al. 2018; Fang et al. 2020; Yip et al. 2022; Wan et al. 2023). Even a single session of robotic gait training can result in rhythmic muscle activations (Ramanujam, Cirnigliaro, et al. 2018). However, it is crucial to understand cerebral activity post‐SCI and recognize residual neurological functions during assisted robotic gait. Regarding our motor task design, the robotic gait training, Kim et al. (2016) observed that robot‐assisted gait training, compared to stepping and treadmill walking, facilitated cortical activation in healthy subjects, which could suggest an increase in cortical activity in SCI during this task.

This study aims to evaluate neurophysiological data on the hemodynamic response from the cerebral motor cortex of spinal cord–injured individuals performing an assisted robotic gait task using fNIRS. We hypothesize (1) an increase in the hemodynamic response during gait compared to rest and (2) hemodynamic response variability according to the severity of the injury, as classified by the ASIA scale. With this, we hope to demonstrate that fNIRS could detect residual cortical activity, providing a valuable tool for enhancing the neurological rehabilitation of individuals with SCI.

## 2 Methods

### 2.1 Participants

Inclusion criteria were individuals (1) with incomplete SCI; (2) with severity graded as C or D (according to AIS); (3) with paraplegic or tetraplegic level; (4) with traumatic or non‐traumatic etiology; (5) in the acute (< 12 months) or chronic phase of injury; (6) without association with brain injury from MRI scans, amputation, convulsive syndrome, or cognitive deficit; (7) with absence of pressure ulcer; and (8) with muscle spasticity graded < 3 (MAS: Modified Ashworth Scale). The research protocol was approved by the local university ethics committee (CAAE: 57068022.9.0000.0068) and conducted in agreement with the Declaration of Helsinki.

### 2.2 Task and Equipment

**FIGURE 1:**
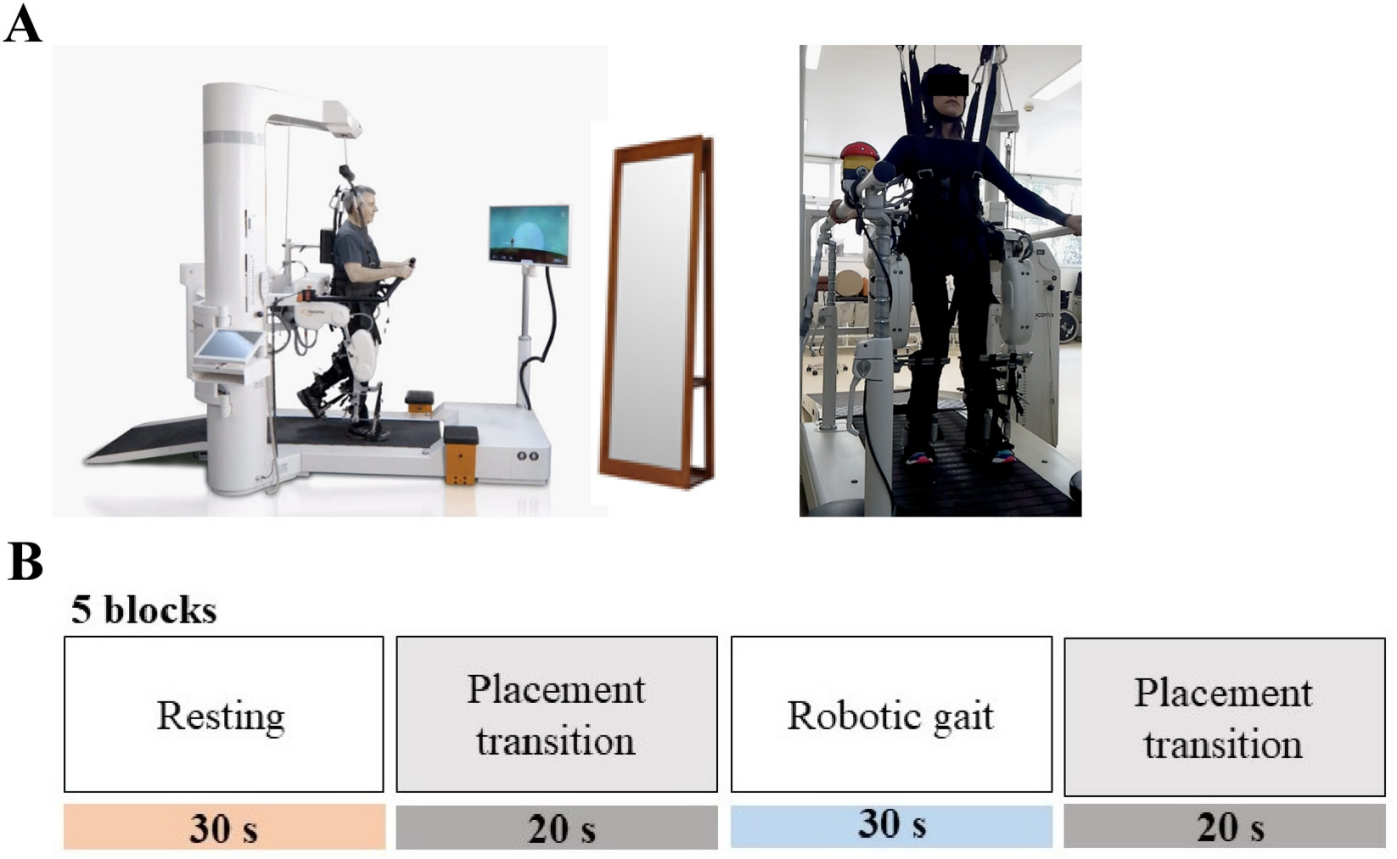
(A) Experimental setup showing participant positioning within the robotic gait device. (B) Flowchart of the experiment showing the five continuous cycles of orthostatic (30 s), Placement Transition 1 (20 s), robotic gait (30 s), and Placement Transition 2 (20 s).

The individuals were positioned inside a robotic gait device (Lokomat, Hocoma), containing a body‐weight support system on a treadmill (BWST) (Figure 1A). Robot‐assisted gait was delivered in a highly assisted mode (substantial body‐weight support [BWT] and robotic guidance force [GF]), which is typical in early neurorehabilitation.

To preset the parameters on the robotic device and to offer physical preparation—aimed at avoiding orthostatic hypotension, excessive spasticity during the stretching mechanism promoted by the robotic legs, and detecting possible discomforts (pelvic and trunk harnesses, leg braces, as well as fear of resuming orthostatic posture and gait)—the individuals performed two sessions on different days of robotic gait training before the procedure with fNIRS motor task: 40 min, TS (treadmill speed) 1.5 km/h, BWT 50%, and GF 70%. During this phase, participants were instructed to remain awake and look forward and to engage in the gait training when possible; however, given the assistance level and injury severity, voluntary motor output could not be technically ensured, quantified, or distinguished from spasticity, commonly present in this type of neurological injury, during the distension of muscle tendons. Therefore, the fNIRS signals during robot‐assisted gait likely reflect a mixture of processes including somatosensory afference, postural/vestibular demands, visual attention, arousal, and (to an unknown extent) motor intent. In the third session, the neurophysiological profile using fNIRS was evaluated, and the motor instructions were adapted.

The individuals performed the gait task at the robotic gait device, using the same parameters of TS 1.5 km/h, BWT 50%, and GF 70%, during 8 min and 40 s, which included 20 s of initial preparation, followed by five continuous cycles of an orthostatic block (30 s), Placement Transition 1 (20 s), robotic gait block (30 s), and Placement Transition 2 (20 s) (Figure 1B). During the orthostatic block, the individuals had their feet leaning on the treadmill and were instructed to keep their eyes open, with a relaxed mind, while looking at a mirror in front of them. This block was considered a “resting block.” The resting baseline was collected in an upright stance with participants instructed to keep their gaze forward. Accordingly, this condition does not represent a fully “neutral” physiological baseline because upright posture and sustained visual attention can engage cortical resources and autonomic arousal. Therefore, contrasts between robot‐assisted gait and “rest” should be interpreted as changes relative to an upright, visually attended baseline rather than relative to eyes‐closed supine rest. For the robotic gait block, the individuals were instructed to keep a relaxed mind while looking at themselves through the mirror in front of them. They were not instructed to try to produce a voluntary muscle contraction or movement with the lower limbs. Placement Transition 1 refers to lifting the static module containing the patient inside the robotic leg, switching on the robotic leg with the treadmill, and lowering the same module. Placement Transition 2 refers to lifting the moving module, switching off the robotic leg with the treadmill, and lowering the static module. The robotic gait task was performed with support and stabilization of the trunk and with the movement of the robotic leg at the hips and knees in the sagittal plane for flexion and extension. The ankle joint of the subjects was stabilized using fixing strips and kept in a neutral position or with a few degrees of dorsiflexion to avoid joint injuries during walking. This joint remained passive throughout the gait cycle because there was no robotic automation of this joint.

### 2.3 Brain Function Assessment

To evaluate the brain hemodynamic response (an indirect measure of brain function), the concentrations of oxyhemoglobin (oxy‐Hb) and deoxyhemoglobin (deoxy‐Hb) during the robotic gait task were acquired with a NIRSport system (NIRx Medical Technologies, Berlin, Germany) with 16 sources that emit 760‐ and 850‐nm frequency‐modulated wavelengths and 16 receptors. The sampling rate was set at 7.81 Hz. Optodes were placed on a measuring cap based on the 10–5 international system, and the fNIRS Optodes' Location Decider (fOLD) toolbox (Zimeo Morais et al. 2018) and the Brodmann area atlas (Rorden and Brett 2000) were used to define the following regions of interest: supplementary motor area (SMA, Brodmann Area 8 or frontal eye fields, which is also covered by part of the SMA), dorsolateral prefrontal cortex (DLPFC, Brodmann Area 9), primary motor cortex (M1, Brodmann Area 4), and primary somatosensory cortex (S1, Brodmann Area 7). These regions were chosen to explore the relation between a motor preparation area (SMA), a cognitive resolution area (DLPFC), a motor execution area (M1), and a sensorial area (S1). The right primary auditory cortex was used as a control area because it is not expected to be modulated by the implemented task. Due to the limited number of optodes, we could only cover part of these cortical areas. To ensure that the cap was correctly placed on each subject's head, we considered the Cz position as a reference between the nasion and the inion and between the left and right preauricular points. After placing the measuring cap, we also used a thick black overcap to cover the optodes properly, attenuating interference from environmental light.

The fNIRS data were recorded with the NIRStar15‐2 acquisition software and analyzed offline with nirsLAB v2017.06 (http://www.nitrc.org/projects/fnirs_downstate/). Channels with a high noise level were rejected, that is, those with a coefficient of variation (standard deviation/mean) exceeding 7.5% or a gain level equal to 3, following NIRSport specifications. After the conversion of intensity data to optical density, oxy‐Hb and deoxy‐Hb concentration changes were calculated using the modified Beer–Lambert equation (according to parameters from Gratzer WB, Medical Research Council Labs, Holly Hill, London, and Kollias N, Wellman Laboratories, Harvard Medical School, Boston, MA; http://omlc.ogi.edu/spectra/hemoglobin/summary.html). Channel‐wise statistical analysis was conducted using an autoregressively whitened robust regression model embedded in NIRSLab v2017.06 (Barker et al. 2013). This analytical approach deals with physiological noise and motion artifacts statistically within the general linear model (GLM), with no prior data preprocessing. Regressors related to the two types of stimuli presented in each trial were included in the GLM to model the effects of the robotic gait task and resting block. The intervals from placement transition were considered as baseline. For the generation of the regressors, a canonical double‐gamma hemodynamic response function, with a 6‐s time‐to‐peak, was used for convolution with the functions defined by the onsets and durations from each type of stimulus. Further, to attenuate hemodynamic effects unassociated with task‐related brain activity, the signal from the control channel was used as a confound regressor.

Nonparametric statistics were used to analyze the data due to the non‐normality and non‐homogeneity of variances (tested by the Shapiro–Wilk test and the Levene statistic, respectively). First, considering all individuals, the Wilcoxon matched‐pairs test was used to compare blocks (robotic gait × resting). Subsequently, a Mann–Whitney test was performed for the robotic gait block, comparing (1) acute × chronic groups, (2) paraplegic × tetraplegic groups, and (3) ASIA C × ASIA D groups. An alpha level of 0.05 was used for all statistical tests performed in Statistica software. To mitigate the risk of false‐positive findings in the context of a modest and heterogeneous sample, we prespecified a limited set of primary regions of interest based on a priori neurophysiological hypotheses (SMA and M1 oxy‐Hb for the within‐subject block comparison; M1 oxy‐Hb for between‐group comparisons). All other ROI comparisons (DLPFC and S1) were treated as exploratory. Alongside *p*‐values, we now report effect sizes derived from the standardized *Z* statistic for all nonparametric tests to facilitate interpretation.

## 3 Results

Twenty‐three individuals with SCI participated in the study: 17 males, 6 females; age, 38.9 ± 12.3 years; duration of injury, 18.0 ± 18.1 months; 11 acute, 12 chronic; degree of injury, 10 ASIA C, 13 ASIA D; 20 traumatic and 3 non‐traumatic injuries; and 15 paraplegics (level of injury from T5 to L4) and 8 tetraplegics (level of injury from C4 to C8). Individuals were classified into acute (up to 12 months of injury) and chronic (more than 12 months of injury).

**FIGURE 2:**
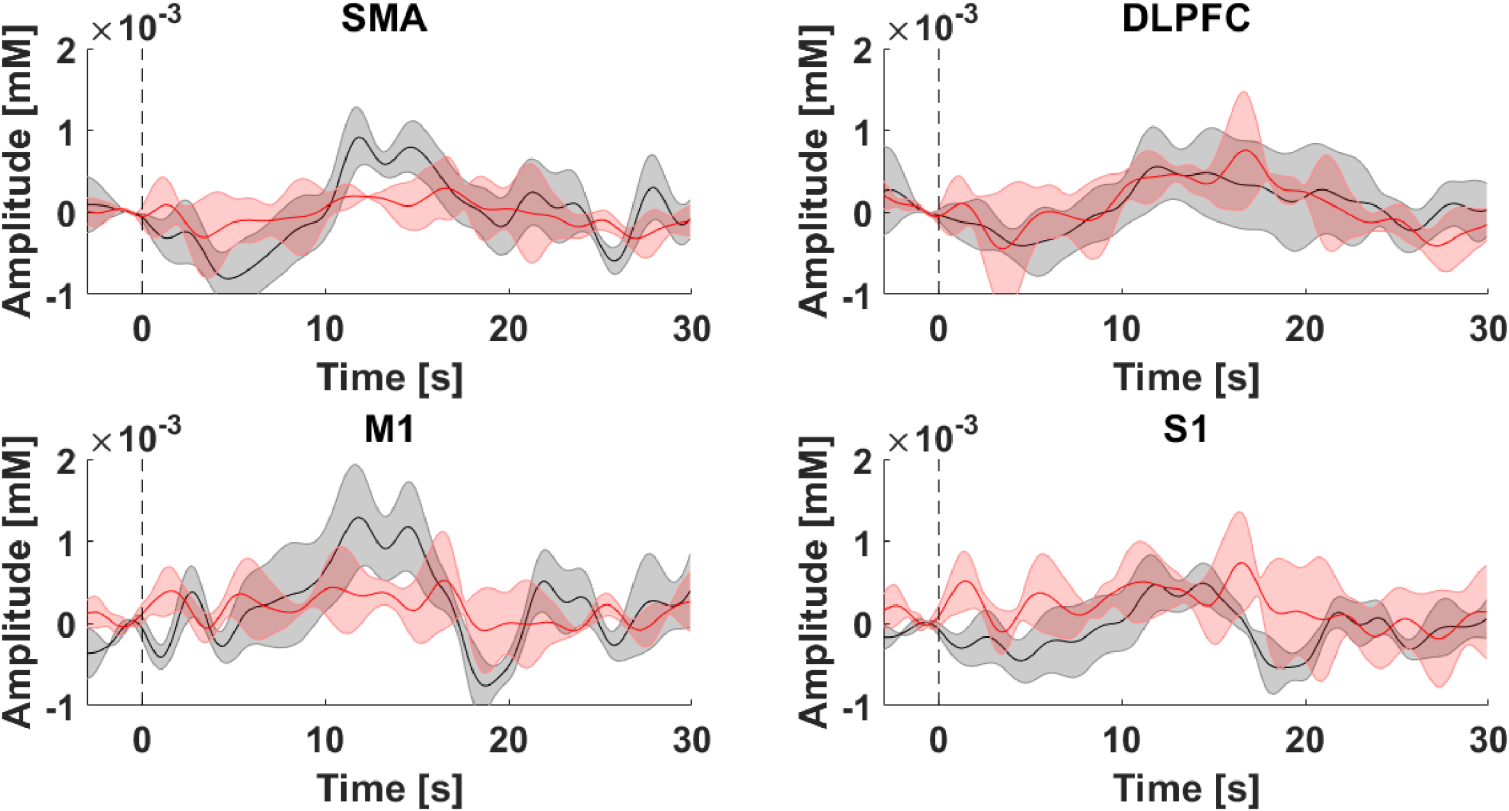
Average values (standard deviation) of hemodynamic response for oxygenated hemoglobin over the supplementary motor area (SMA), the dorsolateral prefrontal cortex (DLPFC), the primary motor cortex (M1), and the primary somatosensory cortex (S1) for robotic gait (black) and resting (red) blocks. A vertical dash indicates when the blocks start.

**FIGURE 3:**
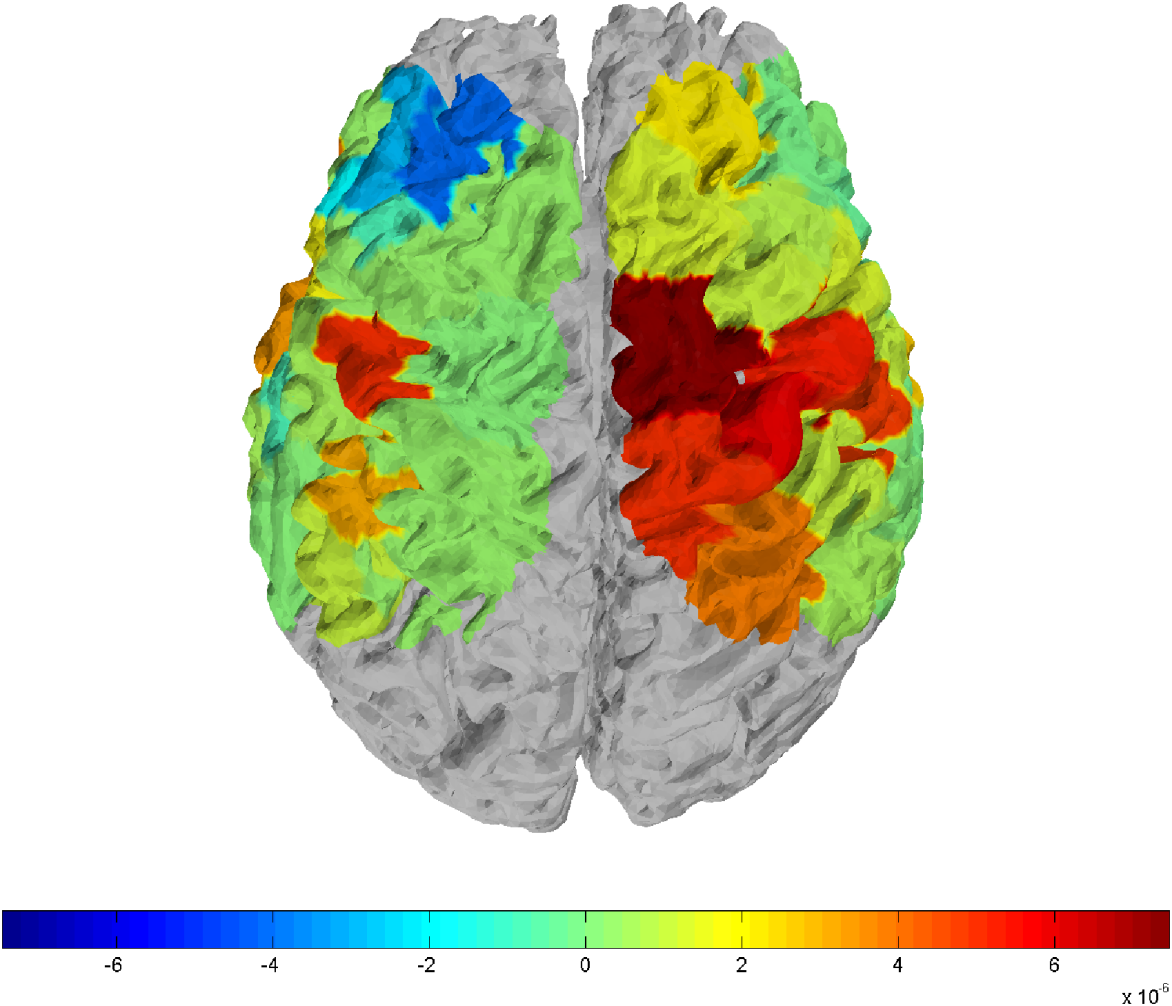
Representative figure of a participant showing the hemodynamic response for oxygenated hemoglobin values.

As the evidence indicates, oxy‐Hb values are more reliable and sensitive to locomotion‐related cerebral blood flow than deoxy‐Hb values (Miyai et al. 2001); only the oxy‐Hb concentration data are presented. The mean and standard deviation of hemodynamic response for oxy‐Hb over SMA, DLPFC, M1, and S1 are shown in Figure 2. Figure 3 shows the hemodynamic response for oxygenated hemoglobin values of one participant.

Considering all individuals, there was a significant difference in SMA (*Z* = 2.43, *p* = 0.015, *r* = 0.507), with higher oxy‐Hb values in the robotic gait task (M = 5.54 × 10^−7^ mM, SE = 1.31 × 10^−5^) compared to resting (M = −2.38 × 10^−5^ mM, SE = 1.43 × 10^−5^). There was a significant difference in M1 (*Z* = 3.72, *p* = 0.001, *r* = 0.776), with higher oxy‐Hb values in the robotic gait task (M = 4.64 × 10^−5^ mM, SE = 2.53 × 10^−5^) compared to resting (M = −2.12 × 10^−5^ mM, SE = 1.14 × 10^−5^). There was no significant difference between DLPFC (*p* = 0.083) and S1 (*p* = 0.059).

For the comparison between acute and chronic groups, there was a significant difference in M1 (*Z* = −2.45, *p* = 0.014, *r* = 0.511), with higher oxy‐Hb values in the chronic group (M = 3.63 × 10^−5^ mM, SE = 1.58 × 10^−5^) compared to the acute group (M = 3.51 × 10^−6^ mM, SE = 2.81 × 10^−6^). There was no significant difference for DLPFC (*p* = 0.372), SMA (*p* = 0.310), and S1 (*p* = 0.559).

Table 1 shows the clinical characteristics of the individuals according to the classification of acute (up to 12 months of injury) and chronic (more than 12 months of injury).

**TABLE 1:** Individual characteristics considering the classification between acute and chronic injuries.

	Acute (*n* = 11)	Chronic (*n* = 12)
Sex	8 M/3 F	9 M/3 F
Age (years)	37.6 ± 12.6	40.0 ± 12.3
Duration of injury (months)	7.2 ± 2.8	27.9 ± 20.6
ASIA score (A–E)	3 C/8 D	7 C/5 D
Paraplegic/tetraplegic	6/5	9/3

For the comparison between paraplegic and tetraplegic groups, there was a significant difference in M1 (*Z* = 2.64, *p* = 0.008, *r* = 0.550), with higher oxy‐Hb values in the individuals with paraplegia (M = 6.73 × 10^−5^ mM, SE = 4.54 × 10^−5^) compared to the individuals with tetraplegia (M = 1.54 × 10^−6^ mM, SE = 2.25 × 10^−6^). There was no significant difference for DLPFC (*p* = 0.138), SMA (*p* = 0.093), and S1 (*p* = 0.245).

For the comparison between ASIA groups, there was no significant difference for DLPFC (*p* = 0.852), SMA (*p* = 0.321), M1 (*p* = 0.166), and S1 (*p* = 0.709). Table 2 shows the clinical characteristics of the individuals according to the classification of paraplegic and tetraplegic.

**TABLE 2:** Individual characteristics considering the classification between paraplegic and tetraplegic injuries.

	Paraplegic (*n* = 15)	Tetraplegic (*n* = 8)
Sex	11 M/4 F	6 M/2 F
Age (years)	35 ± 10.8	46.2 ± 12.0
Duration of injury (months)	15.8 ± 10.0	22.2 ± 28.2
ASIA score (A–E)	9 C/6 D	1 C/7 D

## 4 Discussion

This study aimed to determine whether activity in cortical areas, formerly in control of below‐lesion movements, could still be observed using fNIRS in chronic SCI individuals while performing a robot‐assisted gait task. Our results show greater activity in the motor preparation area (SMA) and motor execution area (M1) during robotic gait tasks than resting. Our results suggest that, despite the motor severity inherent in the lesion profile, the motor stimulus in the lower limbs, in the context of assisted walking, proved important in activating the cerebral cortex's motor areas. Besides, comparing the acute and chronic groups, we observed more activity in motor area M1 in the chronic SCI group during the robotic gait task. Moreover, individuals with paraplegia exhibited greater activity in M1 during the robotic gait task than those with tetraplegia.

Our results showed no differences between ASIA C and ASIA D. It is well known that changes in ASIA grade may not necessarily indicate meaningful changes in the ability to perform activities of daily living. As suggested in a multicenter study with traumatic SCI (Van Middendorp et al. 2009), the isolated use of the ASIA evaluation may not reflect the prognosis of the ability to walk.

Interestingly, using fNIRS, Koenraadt et al. (2014) observed preserved foot motor cortex activity in six complete SCI individuals attempting foot tapping movements. In another fNIRS study with 14 healthy individuals, Kim et al. (2016) noted increased cortical activation in the SMA and M1 during robot‐assisted walking. Our study is the first to show, through fNIRS analysis, the preservation of motor cerebral cortical areas during assisted gait in SCI individuals. The cortical areas that exhibited the most activity were the motor areas, SMA and M1, which correspond directly to the type of task performed, as they are responsible for postural movements, axial (trunk) and proximal muscles (SMA), and more distal movements requiring greater skill and selectivity (M1). The task was carried out in a passive context, with the movement of the lower limbs conducted by the robotic legs of the gait device, leading to the hypothesis that the absence of more complex cognitive engagement in terms of motor planning explained the lesser recruitment and participation of the DLPFC area during the task (Crossman 2024).

Concerning time since SCI, our results show an increase in oxy‐Hb in the chronic phase compared to the acute phase. We hypothesize that the increase in hemodynamic motor brain activity in the chronic phase would indicate that motor ability is facilitated postinjury, which was already observed in past studies (Kokotilo et al. 2009). Studies with fMRI have shown preservation of motor cortices representation in the acute and chronic (Alkadhi et al. 2005; Cramer et al. 2005; Enzinger et al. 2008; Jurkiewicz et al. 2010; Hotz‐Boendermaker et al. 2011) phases of SCI. These studies support preserving numerous aspects of motor system functioning in the brain following long‐term SCI, though with reduced intensity and heightened variability. Jurkiewicz et al. (2007) analyzed brain activity during wrist extension through subsequent fMRI evaluations in individuals from 1 week to 1 year after their cervical SCI. They found reduced activation within the M1 in the subacute phase, followed by a progressive increase in M1 activation in the chronic phase after the SCI. Similarly, an expansion of M1 activity was observed in an EEG study after training with the BWST system, in a group of eight individuals with complete paraplegic SCI (Donati et al. 2016), aligning with our findings of greater M1 activity during the robotic gait task among paraplegic individuals.

Furthermore, in our study, SCI individuals looked at themselves through a mirror while performing the robotic gait task. Studies have shown that motor sequence learning in healthy subjects occurs through movement observation (Cross et al. 2009). Although there is a lack of studies concerning mirror therapy and gait training in human SCI, a meta‐analysis addressed the role of mirror therapy in improving lower limb motricity in stroke subjects and observed that this strategy might have a positive effect (Broderick et al. 2018). Our results suggest that observing their body movement may have positively influenced the activation of the motor cortical areas in our study, even after an extended period of lower limb disuse.

In our scenario, the subjects were not engaged in trying to perform voluntary muscle contractions with their lower limbs following the robotic legs' movement. Therefore, analyzing the neurophysiological profile in this less complex condition is fundamental to understanding the starting point or a more basic stage for the activation of the motor cortex. Subsequently, this will enable us to evaluate and comprehend other conditions involving greater technical complexity of training and more severe SCI.

Walking is widely recognized for its positive effects on cognitive function, with evidence suggesting that these activities stimulate brain health and can slow cognitive decline. This concept extends to individuals with SCI, for whom maintaining physical activity, including assisted walking when feasible, could be especially beneficial. For SCI, assisted walking technologies, such as robotic gait devices, have shown promise in improving physical health and potentially mitigating cognitive decline by promoting sensory input and motor activity that stimulate brain function (Winchester et al. 2005). Furthermore, a study by Hicks et al. (2011) on the role of exercise in SCI individuals highlighted the importance of regular physical activity in maintaining cognitive function, suggesting that exercise could provide neuroprotective benefits and enhance cognitive reserve. The role of M1 activity extends beyond physical rehabilitation to include cognitive and sensory functions, underscoring the interconnectedness of motor activity and cognitive health. Physical exercises that stimulate M1 activity also promote cognitive resilience, highlighting the importance of motor area engagement in comprehensive rehabilitation strategies (Ploughman 2008). Our findings support that maintaining walking practice can offer significant benefits in resisting cognitive losses in individuals with SCI.

This study is cross‐sectional and was not designed to infer training‐induced neuroplastic changes or causal effects of robot‐assisted gait on recovery. In addition, we did not test associations between fNIRS‐derived cortical measures and functional gait outcomes (e.g., overground walking capacity, balance measures, or clinical mobility scales). Therefore, statements about “cortical plasticity” or “motor reorganization” should be interpreted as evidence of cortical engagement during assisted stepping rather than as proof of meaningful, therapy‐driven reorganization. Longitudinal and intervention studies that pair graded active participation with concurrent functional outcomes are required to determine whether the observed cortical responses predict or mediate motor recovery. In line with this cautious framing, recent evidence synthesizing clinical trials in motor‐incomplete SCI suggests that robot‐assisted gait training is not consistently superior to conventional physiotherapy for gait‐quality outcomes and that benefits—when present—may depend on injury phase, training dose, and the integration of robotic practice with task‐specific overground therapy (Fabbri et al. 2023). These considerations underscore the need to link neurophysiological measures to clinically meaningful walking performance in future studies.

The sample size is modest and includes clinically heterogeneous participants (acute vs. chronic stage, paraplegia vs. tetraplegia, and AIS C vs. D), yielding unbalanced subgroups. Accordingly, the study is best interpreted as an exploratory, hypothesis‐generating analysis rather than a definitive estimate of subgroup‐specific effects. Because participants were recruited from a specialized rehabilitation setting with strict inclusion/exclusion criteria and a priori power calculation was not feasible at the design stage, we therefore emphasize effect sizes and cautious interpretation. The robot‐assisted gait condition was intentionally delivered with substantial BWT and GF to ensure safety and feasibility across participants with SCI. Under such highly assisted conditions, cortical hemodynamic changes may predominantly reflect sensory afferent processing (including proprioceptive and cutaneous input), balance/postural set, visual attention, and arousal, rather than isolated motor planning or execution. Thus, the observed activation should be interpreted as “cortical engagement during assisted stepping” and not as a direct surrogate of voluntary motor output. Future work should quantify and manipulate active participation (e.g., graded guidance/BWS, torque/EMG‐based effort metrics, or intention‐driven control) to dissociate sensory‐driven from motor‐intent–driven cortical responses. The “rest” condition was performed in an upright posture with sustained forward gaze, which can recruit attentional and postural control networks and is therefore not a true zero‐activation baseline. This choice was made to match posture, head position, and visual input across conditions and to reduce motion‐related artifacts; however, it likely reduces contrast magnitude and may introduce residual confounding related to attention/arousal. Consequently, the reported task‐related changes should be viewed as differences relative to an upright, visually attended baseline. Future studies could include additional baselines (e.g., seated/supine eyes open and eyes closed) or incorporate physiological covariates (e.g., heart rate and respiration) to further separate task‐specific cortical responses from nonspecific arousal.

Our study demonstrates that in chronic SCI and individuals with paraplegia, robotic gait devices can activate neural systems involved in movement planning and control. Given the cross‐sectional design and the absence of functional outcome correlations, these findings should not be interpreted as evidence of therapy‐induced plasticity or as proof of improved motor recovery. Our results indicate five potential applications in neurological and physical rehabilitation: (1) The cortical engagement of brain motor cortex areas occurs after a distant injury to the spinal cord, even in the chronic phase after SCI. (2) The presence of activity in the brain motor cortex during the execution of a motor task specifically related to walking reinforces the importance of complementing the analysis of residual neurological functions in patients with SCI. (3) As an assessment method, fNIRS, along with imaging exams that analyze the anatomical integrity of neurological structures, can assist in achieving a more comprehensive and accurate diagnosis and prognosis. (4) The preservation of motor planning and execution could be used in BCI applications to operate a robotic device within an assistive and neurological recovery context. (5) The utilization of movement observation, such as mirror therapy, has the potential to serve as a valuable supplementary tool within the field of neurorehabilitation.

## Author Contributions

**Ana Rita C. Donati:** conceptualization, data curation, investigation, validation, visualization, writing – original draft. **Daniel Boari Coelho:** conceptualization, formal analysis, software, writing – original draft. **João Ricardo Sato:** formal analysis, methodology, validation, writing – original draft. **Felipe Fregni:** supervision, validation, writing – review and editing. **Linamara Rizzo Battistella:** funding acquisition, project administration, supervision, writing – review and editing.

## Funding

This work was supported by the Fundação de Amparo à Pesquisa do Estado de São Paulo (2017/12943‐8) and the Ministério da Saúde (25000.160.761/2014‐54).

## Conflicts of Interest

The authors declare no conflicts of interest.

## Supporting information


**Data S1:** Supporting Information.

## Data Availability

The data are in the supporting information. More information will be provided by the corresponding authors.
